# Retraction: Relationship Between Chronic Kidney Disease Staging and Vitamin D Deficiency: A Retrospective Study

**DOI:** 10.7759/cureus.r55

**Published:** 2022-03-17

**Authors:** Theodosios Kantas, Camilo Andrés Avendaño Capriles, Sabir Babor, Tenzin Tamdin, Hady Al-Rihani, Anusha Thalla, Ahmed Adel Abdelmawla, Fares Mohammed Saeed Muthanna, Sohaib Tousif

**Affiliations:** 1 Surgery, Elpis - General Hospital of Athens, Athens, GRC; 2 Foundations of Clinical Research (FCR) Program, Harvard Medical School, Boston, USA; 3 Medicine, Universidad del Norte, Barranquilla, COL; 4 Medical School, Poznan University of Medical Sciences, Poznan, POL; 5 Medical School, Central America Health Sciences University, Ladyville, BLZ; 6 Surgery, Rafik Hariri University Hospital, Beirut, LBN; 7 Medicine, Katuri Medical College, Guntur, IND; 8 Department of Medicine, Aim Shams University, Cairo, EGY; 9 Pharmacy, Walailak University, Nakhon Si Thammarat, THA; 10 Medical School, Ziauddin Medical University, Karachi, PAK

This article has been retracted due to the unknown origin of the data, lack of verified IRB approval, and purchased authorships. While not listed as an author, it was discovered that Rahil Barkat wrote and coordinated the submission of this article. Mr. Barkat was involved in data theft and misuse in two recently published Cureus articles, which have since been retracted.

As the origin of this article’s data and verified IRB approval cannot be confirmed, we have made the decision to retract this article. Cureus has confirmed that the co-authors were asked by Mr. Barkat to proofread the article and provide payment in exchange for authorship. (Proofreading is an insufficient contribution to warrant authorship as defined by ICMJE.) These payments were made in the guise of “editing fees” but greatly exceed any editing fees paid to Cureus. While these authors may have been defrauded by Mr. Barkat, they remain complicit due to their lack of honest contributions to the article.

